# Supplementary material for: The genome sequence of a braconid wasp, Microplitis deprimator (Fabricius, 1798)
Source: Wellcome Open Res. 2024 Nov 5;9:651. [Version 1] doi: 10.12688/wellcomeopenres.23322.1 (PMC11803444; doi:10.12688/wellcomeopenres.23322.1)
Supplement: Peer review history

# Reviewer report: Reviewer response for version 1

**DOI:** 10.21956/wellcomeopenres.25705.r114357

**Published:** 2024-12-24

**Authors:** Cheng Sun

**Affiliations:** 1 Capital Normal University, Beijing, China

The authors provided a high-quality, chromosome-level of genome assembly for braconid wasp, *Microplitis deprimator* (Arthropoda; Insecta; Hymenoptera; Braconidae), which is valuable in understand the evolution and biology of related wasps. In general, the Methods were sound and Results were reliable. I will suggest the authors to make the genome annotation results available for readers, along with the publication of the paper.

Are sufficient details of methods and materials provided to allow replication by others?

Yes

Is the rationale for creating the dataset(s) clearly described?

Yes

Are the datasets clearly presented in a useable and accessible format?

Yes

Are the protocols appropriate and is the work technically sound?

Yes

Reviewer Expertise:

genome evolution

I confirm that I have read this submission and believe that I have an appropriate level of expertise to confirm that it is of an acceptable scientific standard.

# Reviewer report: Reviewer response for version 1

**DOI:** 10.21956/wellcomeopenres.25705.r112457

**Published:** 2024-12-20

**Authors:** Terrence Sylvester

**Affiliations:** 1 University of Memphis, Memphis, TN, USA

The authors present the genome sequence of the braconid wasp, *Microplitis deprimator*. The authors have done an excellent job of generating the genome assembly and scaffolding it up to chromosome level. The authors follow a standard and widely accepted genome sequencing and assembly pipeline. Data are well presented and publicly available. I do not have any significant remarks on the work presented in this manuscript.

Are sufficient details of methods and materials provided to allow replication by others?

Yes

Is the rationale for creating the dataset(s) clearly described?

Yes

Are the datasets clearly presented in a useable and accessible format?

Yes

Are the protocols appropriate and is the work technically sound?

Yes

Reviewer Expertise:

genomics

I confirm that I have read this submission and believe that I have an appropriate level of expertise to confirm that it is of an acceptable scientific standard.
